# Approaching Authenticity Issues in Fish and Seafood Products by Qualitative Spectroscopy and Chemometrics

**DOI:** 10.3390/molecules24091812

**Published:** 2019-05-10

**Authors:** Sergio Ghidini, Maria Olga Varrà, Emanuela Zanardi

**Affiliations:** Department of Food and Drug, University of Parma, Strada del Taglio 10, 43126 Parma, Italy; sergio.ghidini@unipr.it (S.G.); emanuela.zanardi@unipr.it (E.Z.)

**Keywords:** fish and seafood, food authentication, chemometrics, fingerprinting, wild and farmed, geographical origin, vibrational spectroscopy, absorption/fluorescence spectroscopy, nuclear magnetic resonance, hyperspectral imaging

## Abstract

The intrinsically complex nature of fish and seafood, as well as the complicated organisation of the international fish supply and market, make struggle against counterfeiting and falsification of fish and seafood products very difficult. The development of fast and reliable omics strategies based on spectroscopy in conjunction with multivariate data analysis has been attracting great interest from food scientists, so that the studies linked to fish and seafood authenticity have increased considerably in recent years. The present work has been designed to review the most promising studies dealing with the use of qualitative spectroscopy and chemometrics for the resolution of the key authenticity issues of fish and seafood products, with a focus on species substitution, geographical origin falsification, production method or farming system misrepresentation, and fresh for frozen/thawed product substitution. Within this framework, the potential of fluorescence, vibrational, nuclear magnetic resonance, and hyperspectral imaging spectroscopies, combined with both unsupervised and supervised chemometric techniques, has been highlighted, each time pointing out the trends in using one or another analytical approach and the performances achieved.

## 1. Introduction

The demand for fish and seafood products has increased notably during the last years, mostly as a consequence of the new special attention paid by consumers towards healthier food. The technological development that has invested the whole fisheries sector has additionally contributed to overcome the well-known obstacles to export fish and seafood worldwide, deriving from the high vulnerability of the products, to the point that today more than 35% of all caught and cultured fish is traded across national boundaries [1]. The growing competitiveness of the sector and diversification in fish supply chain have, in turn, led to the presence of a huge variety of look-alike products on the international market, whose global quality features are, however, quite different. More than 700 different species of fish, 100 of molluscan, and 100 of crustacean are, in fact, used as food for humans [2].

In this scenario, what is remarkable is that consumers demand not only for more fish, but for even safer and higher-quality fish, whilst the deliberate or accidental lack of transparency about the identity of products and fraudulent or negligent activities continue to grow. Based on what has been recently reported by the Food and Agriculture Organization, fish and related products have become among the most vulnerable to fraud category of food. Nevertheless, the effective monitoring of illicit practices in the fisheries sector is hampered by the increasing spread of highly processed fish products, in which the presence of different types of fraud can be hidden with ease [3].

The voluntary substitution of commercially valuable fish species with lower quality ones, represents the most recurrent form of fish fraud, although substitution can also take place accidentally when species look so similar that they are mistaken for each other. The geographical provenance and the production process are other current authenticity topics concerning fish and seafood products, whose falsification which is hard to bring to light, has a negative economic impact. Despite being economically motivated, mislabelling concerning these issues may occasionally represent a risk to public health. The illegal commercialisation of poisonous fish species (*Tetraodontidae, Molidae, Diodontidae*, and *Canthigasteridae* families) or the replacement of certain kinds of raw fish fillets with gastro-intestinal toxic fish (i.e., those belonging to the *Gempylidae* family) are just some of many examples. Likewise, occurrence of some harmful marine biotoxins may be linked to the geographical distribution of the producing organisms [4], while the presence of higher levels of heavy metals or residues of antibiotic and pesticides are more likely to be found in farmed products than in wild ones [5,6,7].

Ensuring a clear discrimination of the authenticity of fish and seafood is of special concern today not only for consumers, but also for producers, traders, and industries. Traceability throughout the whole production chain and at all stages of the market, covered by Regulations 178/2002/EC [8], 1005/2008/EC [9], and 1224/2009/EC [10], is considered to be the starting point for the assurance of a high level of safety and quality of food and ingredients, as it represents the basic instrument not only for preventing illegal activities, but also for protecting consumers through the opportunity to access information about the exact nature and characteristics of fish. Specific regulations for the provision of information to consumers [11], and the requirement to uniquely identify fish and seafood on the label [12], play also an essential role in providing more transparency regarding the nature of the products, as they allow consumers to make informed choices and further contribute to the implementation of seafood traceability. As a matter of fact, labels of all unprocessed and some processed fishery and aquaculture products must include information on both the commercial and scientific names of the species, whether the fish has been caught or farmed, the catch or the production area, the fishing gear used, whether the product has been defrosted, and the date of minimum durability (where appropriate). Many other voluntary claims can also be reported on the label, including the date of catch/harvest for wild/aquaculture products, information about the production techniques and practices, and environmental and ethical information [12].

All the claimed declarations appearing on the label must always be checked to verify whether they are truthful. Therefore, in spite of the utility of the traceability system, the fisheries sector needs effective methods to address the problem of fish authenticity and ensure product quality. Innovative analytical approaches based on the evaluation of total spectral properties, are rapidly gaining ground at all levels of current food authenticity research, thanks to their ability to simultaneously provide lots of information related to physical and chemical characteristics of the food matrix. Recent advances in chemometrics, moreover, have represented a major turning point in the dissemination of ‘fingerprinting strategies’, as they allow for the study of all the genetic, environmental, and other external factors influencing food identity, and to bypass many obstacles related to the application of conventional techniques [13]. This way, chemometrics can be now considered an essential tool for differentiation of similar samples according to the authentication issues of interest.

Until now, several spectroscopic techniques in conjunction with chemometrics have been used as rapid, simple, and cheap tools for fish quality and authenticity testing. Among these, vibrational (near-infrared (NIR), mid-infrared (MIR), Raman), fluorescence or absorption ultraviolet-visible (UV–Vis), and nuclear magnetic resonance (NMR) spectroscopies, together with hyperspectral imaging (HSI) spectroscopy, represent the most used techniques, even if they are still being developed.

Based on this background, the present review article has been designed to highlight the uses and developments of fast and reliable omics strategies based on UV–Vis, NIR, MIR, Raman, NMR, and HSI spectroscopies, with the attempt to address the key authenticity challenges within the fish and seafood sector. To this end, a brief discussion concerning basilar concepts underlying these techniques has been provided, and has been accompanied by a short overview about the implementation of several chemometric tools, in order to highlight the potential benefits in extracting relevant information from spectral data.

The main body of this review focuses specifically on the application, over the years, of spectroscopy and chemometrics to distinguish products in accordance with the species, production method (wild or farmed), farming system (conventional or organic; intensive, semi-intensive, or extensive), geographical provenance (different FAO areas and countries of origin), and the processing technique (fresh or fresh/thawed) that at present, correspond to the key authenticity concerns for which there must be ongoing and effective monitoring.

## 2. A Conceptual Framework of Spectroscopy and Chemometrics

Spectroscopy is the study of electromagnetic radiation interacting with matter, which can be absorbed, transmitted, or scattered on the basis of both the specific frequency of the radiation and the physical/chemical nature of the matter. When absorbed, radiation leads to a change in the energy states of atoms, nuclei, molecules, or crystals that make up matter, inducing an electronic, vibrational, or rotational transition, depending on the energy of the incident radiation [14]. When the radiation, at a specific frequency, is scattered by molecules (as in Raman spectroscopy), some changes can occur in the energy of the incident photon, which transfers parts of its energy to the matter. In any case, the result of these interactions is a spectrum enclosing many features of the matter analysed, which, when properly interpreted with the help of chemometrics, can be used in a great number of different applications. In choosing the most appropriate spectroscopic method to be used, consideration should be given to some factors, which go beyond the purely analytical purposes: the physical state and chemical composition of the sample, sensitivity, specificity, and overall accuracy of the technique, scale of operation, time of analysis, and cost/availability of the instrumentation [15].

For the sake of conciseness, the main features related to spectroscopic techniques used mostly in the food authentication field are summarised in Table 1.

### 2.1. UV–Vis Absorption and Fluorescence Emission Spectroscopy

UV–Vis spectroscopy involves the electronic excitation of molecules containing specific chromophore groups, which results from the absorption of photons at two wavelength regions of the electromagnetic spectrum. In the absorption mode, the amount of light retained by the sample is measured, while in the fluorescence mode the amount of light emitted after absorption is taken into consideration [15]. Typically, the UV–Vis spectrum is characterised by broad absorption or emission peaks which reflect the molecular composition of the matrix: by exploiting the unicity absorption or emission patterns of the entire spectrum, or by measuring the absorbance or fluorescence intensity of the analyte at one wavelength, this spectrum can be used for many food analytical qualitative and quantitative applications, respectively [16,17].

### 2.2. IR Spectroscopy

Infrared spectroscopy involves three different sub-regions of the electromagnetic spectrum, namely NIR, MIR, and FIR, whose absorption by samples results in vibrations of atoms in molecular bonds [18]. These vibrations give out a great amount of information related not only to chemical bonding, but also to the general molecular conformation, structure, and intermolecular interactions within the sample [19]. This way, IR spectra enclose the total sample composition, whose pattern of peaks distribution represents a unique signature profile and whose intensity of bands is linked to the concentration of specific compounds [20,21].

The NIR spectrum of food samples results from absorption by molecular bonds containing prevalently light atoms and it is characterised by the presence of broad and overlapping overtone and combination bands [22,23]. By contrast, spectral signature in the MIR region is characterised by the presence of more intense and delineated bands, whose position and intensity are more informative of molecule’s concentration in the sample [24,25]. Here too, the spectral profile is complex and data mining is very difficult without the use of multivariate data analysis. Finally, with reference to FIR spectroscopy, it is noted that no applications to food authentication are currently available since it relates to molecules containing halogen atoms, organometallic compounds, and inorganic compounds, whose interest is more limited within the context of food research [26].

### 2.3. Raman Spectroscopy

Raman spectroscopy is a molecular vibration technique based on the inelastic Raman scattering, a physical effect that comes with molecular vibrations and triggers a change in the polarizability of the molecule [27]. In particular, this kind of spectroscopy focuses on the measurement of those small fractions of the radiation which is scattered by specific categories of compounds at higher or lower frequencies than incident photons. The typical Raman spectrum, showing intensities of the scattered light versus the wavelengths of the Raman shift, is characterised by sharp and well-resolved bands, which provide information about molecular structure and composition of the matter analysed.

For a long time after its discovery, Raman spectroscopy has been poorly exploited in food applications, by reason of several analytical disadvantages and interference (see Table 1). These drawbacks have now been overcome thanks to the overall technological improvement of Raman equipment: by way of example, surface-enhanced Raman spectroscopy (SERS) has recently made it possible to surmount hurdles related to faint scattering signals [28].

### 2.4. Hyperspectral Imaging

HSI is a technique cobbling together spectroscopy and computer vision to give useful information concerning the physicochemical characteristics of samples in relation to their specific spatial distribution. Briefly, HSI systems provide several hyperspectral images of the tested sample, corresponding to three-dimensional data containers, of which each sub-image is a map showing spatial distribution of the sample constituents in relation to each single wavelength [29,30].

Over the recent years, the steady usage growth of HIS technology in the field of food research has been mainly driven by the availability of different instrumental configurations that exploit fluorescence, absorbance, or light scattering phenomena. On the other side, application of spectral imaging technologies is not at all widespread in the food industry, due to a variety of factors ranging from high costs and low availability of instrumentations, to the computation speed and necessity of expertise by users [31].

### 2.5. NMR Spectroscopy

NMR spectroscopy is a very versatile technique for food analysis and its untargeted applications have become very popular. The first reason for NMR popularity is that the composition of the matter under study can be perfectly mapped out by the overall NMR spectral profiles, thus giving a comprehensive view for the identification of all major and minor food components [32]. At the same time, the area of the NMR spectral bands is directly proportional to the number of nuclei that produce the signal, so the technique is also well-suited for quantitative purposes. Additionally, despite relatively high NMR equipment costs and spectra interpretation difficulties, NMR spectroscopy is one of the only techniques available that can provide information about the regio/stereo chemistry of molecules [33].

On the basis of the physical state of the matter and on the intended aim of NMR application, different methodologies involving the use of NMR have been optimized. Among these, high-resolution NMR, low-field NMR, solid-state NMR, liquid-state NMR, and NMR imaging are the most used ones, any of which requires specific instrumentation and different approaches to sample preparation, data acquisition, and processing [34].

### 2.6. Qualitative Chemometric Methods

Raw spectra resulting from spectroscopic analyses are usually characterised by broad and unresolved bands containing too much information, some of which are certainly useful and need to be retained, but some of which hamper the correct data interpretation and need to be removed. Recent advances in chemometrics have marked an important milestone in spectra analysis, since they have simplified the identification of hidden interrelations between variables providing the key for discrimination and classification of samples [20,35]. In other words, qualitative chemometrics methods help to recognise similarities and dissimilarities within spectral data, which can be used to confirm the authenticity or detect adulteration of food samples [36].

Based on the explorative or predictive nature of the methodology, qualitative chemometric techniques are usually classified into unsupervised and supervised techniques. While unsupervised techniques are independent of prior knowledge of class membership of samples to perform classification, supervised techniques call for such knowledge. Brief descriptions of the principles behind the chemometric techniques which are being used to a greater extent are provided below.

#### 2.6.1. Spectral Pre-Treatments

Pre-treatment of spectral data is recognized as being fully integrated into the chemometric set-up itself. Prior to the development of chemometric models, raw spectroscopic data are suggested to be pre-processed by applying some corrections, aimed to enhance spectral properties and minimize the fraction of systematic variation which does not contain relevant information to the discrimination of samples. One such systematic variation is the sum of different physical effects which arise during instrumental acquisition of spectra (e.g., light scattering or background fluorescence phenomena), which are responsible for the appearance, especially in solids samples, of multiplicative, additive, and non-linearity effects (e.g., overlapping bands, baseline shifts/drifts, random noise) [37].

Thus, pre-processing algorithms are usually classified into signal correction methods (e.g., multiplicative scatter correction, MSC; standard normal variate, SNV), differentiation methods (first, second, or third order derivation), and filtering-based methods (e.g., orthogonal signal correction, OSC; orthogonal wavelet correction, OWAVEC) [38]. While signal correction and filtering-based methods are conceived to retain only the spectral information mainly by suppressing the light-scattering effects, derivative-based methods also help to reduce the spectral complexity through the separation of the broad overlapping bands.

A more detailed description of spectral pre-processing techniques can be widely found in the literature [37,39,40]. Either way, it is essential to point out that spectral filters are most often concatenated to exploit the effects of each one, but this concatenation might increase model complexity and background noise, resulting in an inaccurate chemometric modelling of data and, thus, wrong predictions. For this reason, it is recommended to customize the selection of the pre-treatments prior to performing chemometric analysis according to the spectroscopic technique used and the sample characteristics, trying to restrict, whenever possible, their number.

#### 2.6.2. Unsupervised Methods

Unsupervised methods look at the study of variability among samples for the purpose of identifying their natural characteristics and possible similarities among them, without the need to provide any information about the class to which samples belong.

Between the various available techniques, principal component analysis (PCA) is the most used one. PCA is a quite basic projection method able to reduce the original correlated variables into a smaller number of new uncorrelated latent variables (known as principal components), containing as much systematic variation as possible of the original data [41]. Score plot outputs deriving from PCA applications show in a simple and intuitive graphical way the hidden structures among samples, the interrelations among variables and between samples and variables, the probable presence of any outliers, and possible groupings or dispersion of sample according to specific class membership.

Hierarchical cluster analysis (HCA) is another frequently employed unsupervised method, based on the splitting of samples into different clusters. This splitting is based on the degree of analogy among samples and it is generally performed by evaluating the Mahalanobis or Euclidean distance between the same samples. The hierarchical approach followed is thus aimed at constructing a ladder, in which the most closely related samples are first classified into small groups, and then progressively assembled into bigger groups including less similar samples [35]. Results of HCA are graphically expressed by tree diagrams (dendrograms) showing relationships among clusters; nevertheless, despite being easily computable, dendrograms are often misunderstood, since the number of clusters to be considered is arbitrary, making the interpretation of results more subjective than objective.

#### 2.6.3. Supervised Methods

Supervised techniques require the previous knowledge of the class membership of the samples tested, which can be used to develop predictive models able to discriminate and classify future unidentified samples. There are several different chemometric techniques belonging to the category of the supervised methods, most of which require a training set (to find classification rules for the sample), and a test set (to assess the predictability of the model developed) [42].

Linear discriminant analysis (LDA) and quadratic discriminant analysis (QDA) are variance-based methods which use Euclidean distance to find those combinations of the original variables determining maximum separation among the different groups of samples [20]. Both techniques presume that the measurements within each class are normally distributed, but while LDA supposes that dispersion (covariance) is identical for all the classes, QDA, on the contrary, allows the possibility of different dispersion to be present within different classes [35]. Although QDA is considered an extension of LDA, there are some common limitations, for instance the risks of overfitting and failing in classification, especially when the samples size for each class in unbalanced.

K-nearest neighbors (k-NN) clustering is one of the simplest method to discriminate samples on the basis of the distance among them. After choosing the adequate number of k-neighbor samples, the algorithm identifies the k-nearest samples of known class membership to select the classification of unknown samples. This method, unlike LDA and QDA, does not require any prior assumption and its success is independent of the homogeneity of sample numbers in each tested class [43].

Among supervised machine learning approaches, support vector machines (SVM) are particularly advantageous when samples classification is complicated by non-linearity and high dimensional space. The core of the method is the use of specific functions for pattern analysis (kernel algorithms), through which the margin of separation between classes is maximised and complex classification problems that are not linear in the initial dimension (but may be at high dimensional spaces) are resolved [20].

Similarly, artificial neural networks (ANN) is a machine learning method characterised by the ability to adapt to the data, providing classification also in the presence of non-linearity input–output relationships. Structured and organized in a less complex way than SVM, ANN usually generate a more rapid response at a lower computational cost; these efforts, however, are counterbalanced by a reduction in accuracy [20,44]. Nevertheless, ANN suffers from poor data generalisation and, by consequence, it is inclined to return model’s overfitting errors. This tendency to overfitting is the main reason why accurate ANN computation analyses call for a very high number of samples to be considered, and at the same time, require strict internal and external validations to be performed, where the training set and the test set should enclose as much similar variability as possible [45].

Soft independent modelling of class analogy (SIMCA) is an alternative pattern recognition method which first performs individual PCA on the samples for each class they must be assigned to, in order to compress original variables into a smaller number of new principal components. Principal components and critical distances computed are then used to delineate a confidence limit for each class. Unknown samples are then assigned to the class to which they get close by projection into the resulting multidimensional space [36]. SIMCA is particularly useful when samples belong to several different classes; since maximum class-separation is not covered by the method, the interpretation of the outcomes may be difficult, if not impossible [20].

Regression-based supervised discriminant analyses exploit specific classification algorithms to model the interrelations existing between measured variables (i.e., spectra) and qualitative parameters (i.e., class membership), such that maximum separation between the different groups of samples is achieved. Partial least square-discriminant analysis (PLS-DA) and orthogonal partial least square-discriminant analysis (OPLS-DA) belong to this category of techniques. PLS-DA involves a standard PLS regression to find interrelations between the X-matrix (containing measured variables) and Y-matrix (containing categorical variables) by building new variables (latent variables). These interrelations allow not only to classify new samples into one of the Y-groups based on measured spectrum, but also to identify variables that mostly contribute to the classification. Although PLS-DA has the advantage of modelling noisy and highly collinear data efficiently, the technique is often unsuccessful when the non-related (orthogonal) variability in the X-matrix is substantial, since it hinders the correct interpretation of the results [20]. This drawback can be overcome by the application of OPLS-DA, through which the orthogonal variability within the X-matrix is separated from the related (predicted) variability and then modelled apart. Consequently, if samples cannot be discriminated along the predictive direction, the orthogonal variability may be handled to increase the effectiveness of discrimination among classes [46].

## 3. Authenticating Fish and Seafood through the Application of Qualitative Spectroscopy and Chemometrics

Spectroscopic and chemometric analyses have been used over the years for many applications in fishery research, those in the authentication field being among the most promising ones. Some of the works concerning the flexibility of spectroscopy in fish and seafood analysis have already been reviewed by different authors [24,25,47,48,49], but they have mainly centred on illustration of the advances of the available techniques for quality attributes assessment, as well as on the advantages and limitations of the single type of technique over traditional methods.

**Figure 1:**
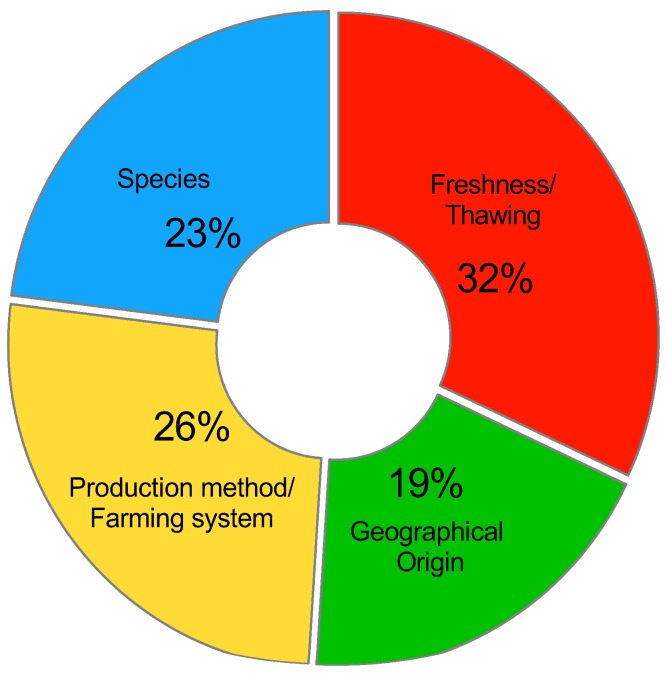
Percentage distribution of the authenticity issues covered by the scientific literature reviewed in the present work. Data were collected in February 2019 from the web search engine Google Scholar (search criteria: time period: “any time”, and keywords: ‘‘fish and/or seafood”; “authenticity”; “spectroscopy”; “chemometrics”.

**Figure 2:**
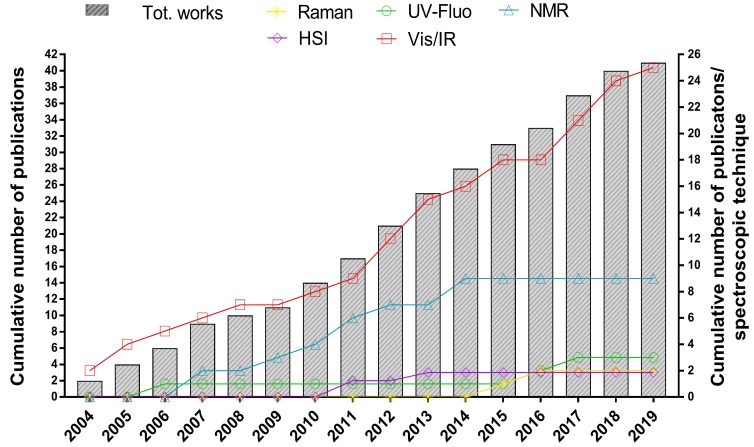
Combined bars and lines graph, where bars (plotted against the left Y-axis) show the cumulative number of scientific works concerning the use of spectroscopy and chemometrics for fish authentication purposes, and lines (plotted against the right Y-axis) show the cumulative number of works using each spectroscopic technique. Data were collected in February 2019 from the web search engine Google Scholar (search criteria: time period: “any time”, and keywords: ‘‘fish and/or seafood”; “authenticity”; “spectroscopy”; “chemometrics”.

Therefore, in the following section, more attention has been paid to the resolution, on a case-by-case basis, of the weightiest authentication issues in the fish and seafood sector, namely species substitution, geographical origin falsification, production method or farming system misrepresentation, and fresh for frozen/thawed product substitution, each time pointing out the trends in using one or another method as well as the discrimination performances achieved, which are considered to be the most intuitive parameters used for chemometric models diagnostics. An overview of the most frequently investigated authentication issues in the fishery sector and the trend of using each spectroscopic technique over the years by the scientific community are plotted in Figure 1 and Figure 2, respectively.

### 3.1. Species Substitution

Substitution or counterfeit of high-value fish species with low-value ones has many quality and safety implications. Therefore, the confirmation of scientific and commercial names declared on the label through the use of rapid and low-cost methods is increasingly popular in food research.

#### 3.1.1. Application of Vibrational Spectroscopy

An early study explored Vis-NIR spectroscopy as a tool to detect the counterfeit of Atlantic blue crabmeat (*Callinectes sapidus*) with blue swimmer crabmeat (*Portunus pelagicus*) in 10% increments, taking into consideration their different commercial values [50]. Qualitative chemometric analysis was performed on 400–2498 nm Vis-NIR spectra (previously subjected to different pretreatments to evaluate the effects on model performance), by means of a full-spectrum PCA and a sequential-spectrum PCA. As a result, both the first derivative-pretreated full spectra and second derivative-pretreated sequential spectra, highlighted a trend of samples towards moving from the left part to the right part of the PCA score plot with increased adulteration levels, but authors identified the sequential approach, using 400–1700 nm second derivative spectra, as being the most informative and, thus, the most suitable approach [50].

Based on the fact that the past several years have seen a sharp rise in the interest towards the portability of instruments, which may provide greater flexibility especially in on-line, in-line, and at-line routine quality control, a study performed by O’Brien et al. (2013), explored the ability of a hand-held NIR spectrometer to give positive results of discrimination between high-value and low-value whole fish and fish fillet species [51]. In particular, the objective was to discriminate between two different species of mullet (red mullet from mullet), cod (winter cod from cod), and trout (samlet from salmon trout). NIR spectra (906–1648 nm) obtained from skin (whole fish) and meat (fish fillets), were first pre-processed and then elaborated by PCA and SIMCA analysis. Successful PCA results were achieved only in separating the whole mullet samples, but the discrimination performances improved significantly also for mullet fillets after the application of the SIMCA analysis. PCA failed to discriminate both whole cod and cod fillets, but here too, SIMCA predictions provided a correct assignment of the tested fish samples. Similar outcomes for samlet from salmon trout were achieved [51]. Thus, although PCA investigation failed, SIMCA supervised analysis clearly outlined the possibility to authenticate high quality fish species which are potentially substitutable with lower-quality alternatives. Still in the context of the use of hand-held and compact NIR devices, a broader attempt to distinguish fillets and patties of Atlantic cod (*Gadus morhua*) from those of haddock (*Melanogrammus aeglefinus*) was recently made [52]. Raw fillets and patties of the two fish species were scanned at 950–1650 nm (by the portable instrument) or at 800–2222 nm (by a benchtop instrument) and after being pre-treated with SNV, MSC, or Savitzky–Golay smoothing (SG) coupled with first or second derivative, they were elaborated by means of supervised LDA and SIMCA analysis. Regardless of instrumentation used, the best LDA models were computed on the MSC spectra of both fillets and patties, since the correct classification rate in the external validation step reached 100% [52]. SIMCA class-modelling strategy obtained 100% correctly classified SNV, SG-first derivative, or SG-second derivative fillets spectra acquired by benchtop NIR, and 100% correctly classified MSC fillets spectra acquired with a portable NIR [52]. As for patties, samples acquired by benchtop NIR and portable NIR were 100% correctly classified when spectra were subjected to SG-first derivative or SG-second derivative, and SNV or MSC, respectively. The worst SIMCA outcomes in prediction for patties and fillets were obtained for SG-second derivative spectra acquired with the portable instrument. Despite these results, no significant differences in the performances of the two instruments tested were found, thus confirming equivalent discrimination powers also in processed product.

Different species of freshwater fish of the Cyprinidae family, namely black carp (*Mylopharyngodon piceus*), grass carp (*Ctenopharyngodon idellus*), silver carp (*Hypophthalmichthys molitrix*), bighead carp (*Aristichthys nobilis*), common carp (*Cyprinus carpio*), crucian (*Carassius auratus*), and bream (*Parabramis pekinensis*), were also investigated by NIR spectroscopy [53]. Fish samples were scanned in the 1000–1799 nm region, MSC pre-treated, and pre-reduced in dimensionality by different methods, including PCA, PLS, and fast Fourier transform (FFT). In this case, LDA models were built by using only nine pre-selected spectra wavelengths from the entire spectrum and results obtained showed a good prediction ability of the adopted strategy: PCA-LDA and FFT-LDA models, in fact, showed 100% accuracy, specificity, sensitivity, and precision, even if most of the information was not taken into account by calculation [53].

Zhang et al. (2017) attempted to classify marine fish surimi by 1100–2500 nm NIR spectroscopy, according to the species by which products were composed, namely white croaker (*Argyrosomus argentatus*), hairtail (*Trichiurus haumela*), and red coat (*Nemipterus virgatus*) [54]. According to results obtained from PCA of the pre-processed spectra, the presence of a well-defined and separated cluster associated with red coat surimi species was observed, but the separation of the other two species of surimi samples was not clear [54]. However, as regards LDA results, 100% correct classification rate for external validation datasets after MSC pre-treatment was achieved, demonstrating once again the greater effectiveness of supervised analyses compared to unsupervised ones.

Species authenticity was also studied by comparing FT-NIR and FT-MIR spectra of red mullet and plaice fillets (higher-value species) to those of Atlantic mullet and flounder fillets (lower-value species) [55]. LDA and SIMCA analysis applied to differently pre-treated NIR and MIR spectra (800–2500 nm and 2500–14,300 nm spectral ranges, respectively), clearly discriminated Atlantic mullet fillets from those of the more valuable red mullet. While LDA gave a 100% correct classification percentage in prediction (irrespective of the spectroscopic technique considered), sensitivity and specificity higher than 70% and 100%, respectively, were calculated for FT-NIR spectra subjected to SIMCA analysis [55]. Poorer, but acceptable, results were obtained for flounder and plaice fillets discrimination: in this case, FT-IR spectroscopy showed the best discrimination power, with a prediction ability higher than 83% and a specificity of 100%.

The usefulness of NIR spectroscopy was explored to identify different fish species used to make fishmeal under industrial conditions. The 1100–2500 nm raw or second derivative NIR spectra of samples containing salmon, blue whiting, and other (i.e., mackerel or herring) fish species were elaborated by PCA, LDA, and DPLS (PLS-DA). Models developed correctly classify, on average, more than 80% of the fish meal samples into the three groups assigned according to the fish species [56].

In contrast to the multiple applications of NIR spectroscopy, only one study explored the discrimination abilities of MIR spectroscopy [57]. This study coupled SG- and SNV-pre-treated MIR spectra (2500–20,000 nm) with chemometrics (PCA) to specifically detect adulteration of Atlantic salmon (*Salmo salar*) mini-burgers with different percentage (from 0 to 100%, in steps of 10%) of Rainbow trout (*Onconrhynchus mykiss*). The resulting 11 formulations of salmon burgers were grouped into 11 distinct clusters, even when the samples were stored for different periods of time before acquisition [57].

Only two applications of Raman spectroscopy concerning fish species authentication are available. The aim of the first study was to discriminate 12 different fish fillets of different species by using pre-treated Raman spectra in the range 300–3400 cm^−1^ (about 3940–33,333 nm) recorded by a Raman spectrometer equipped with a 532 nm laser exciting source [58]. HCA analysis applied to the Raman spectra revealed the presence of three major clusters, one corresponding to fish from the Salmonidae family (rainbow trout and Chum salmon), one corresponding to various freshwater fish (zander, Nile perch, pangasius, and European seabass), and one corresponding to various saltwater fish (Atlantic herring, Atlantic pollock, Alaska pollock, Atlantic cod, blue grenadier, and yellowfin tuna). Within these large clusters, spectra were also grouped according to their species in sub-clusters, with a high degree of accuracy of the spectral classification on species level (95.8%) [58]. Similarly, PCA analysis performed on 5000–50,000 nm Raman spectra (acquired by using a 785 nm laser exciting source) discriminated among horse mackerel (*Trachurus trachurus*), European anchovy (*Engraulis encrasicolus*), Bluefish (*Pomatamus saltatrix*), Atlantic salmon (*Salmo salar*), and flying gurnard (*Trigla lucerna*) samples. In this case, however, the study was less rapid and more elaborate since the spectral acquisition was performed on the previously extracted lipid fraction of fish [59].

#### 3.1.2. Application of NMR Spectroscopy

Muscle lipids of four different species of fish belonging to the Gadoid family, namely cod (*Gadus morhua*), haddock (*Melanogrammus aeglifinus*), saithe (*Pollachius virens*), and pollack (*P. pollachius*), were subjected to ^13^C-NMR spectroscopic analysis of phospholipid profiles, in order to authenticate samples according to the species [60]. As a result, supervised LDA and Bayesian belief network (BBN) performed on the resulting ^13^C-NMR spectral peaks provided 78% and 100% of the correctly classified samples, respectively [60]. Other applications of NMR and chemometrics concerning fish species discrimination were not reported in literature until now. In our opinion, the method should be further explored in view of the several potentials and benefits provided, despite disadvantages deriving from the need of sample preparation prior to analysis.

### 3.2. Production Method and Farming System Misrepresentation

The differentiation of the production method of fish and seafood is another relevant aspect in certifying authenticity and traceability. During the last few years, the wild fish catches have been decreasing compared to the aquaculture production, thus supply of the market in farmed products has been growing very fast. From a compositional and organoleptic point of view, a wild fish is quite different from an aquaculture one, and this diversity is inevitably reflected on the different economic value of the two types of products [61,62,63]. By way of example, wild fish is usually characterised by higher levels of muscle protein, saturated, and polyunsaturated fatty acids, while farmed fish by a higher content of total lipid and monounsaturated fatty acids [64,65]. Consequently, the illegal substitution of higher-value wild fish with lower-value farmed fish is not an uncommon occurrence. Additionally, aquaculture fish consist of a number of high-variable products (i.e., extensively, semi-intensively, or intensively farmed fish, as well as organic or conventional farmed fish), whose final characteristics, since influenced by the husbandry environment and, above all, by the diet, are slight and very difficult to identify. This the reason is why the authentication of the production method (wild or farmed, organic or conventional), but also of the farming system of the aquaculture products is of extreme importance from the standpoint of fraud prevention and transparency towards consumers.

#### 3.2.1. Application of Vibrational Spectroscopy

Among various vibrational spectroscopic methods applied to differentiate production processes and farming systems of fish, NIR is once again the most widely used. No application of UV or Raman spectroscopy, to the best of our knowledge, are currently available.

Ottavian et al. (2012) proposed a comparison between the classification performances of wild and farmed European sea bass obtained by three different NIR spectroscopic/chemometric approaches, and the classification performances obtained using only chemical and morphometric features [66]. The use of 1100–2500 nm raw spectra, WPTER-pre-treated spectra (wavelet packet transform for efficient pattern recognition), or of some parameters predicted by building a regression-based model, were found to be equivalent in terms of predictability assessed by PLS-DA and no differences between classification obtained by these models and classification obtained by using only chemical and morphometric data was observed. Moreover, authors identified (by using the variable influence of projection indexes, VIP) the wavelengths related to the absorbance of fat, fatty acids, and water as most influential in differentiating the production process of the fish tested.

More recently, the systems behind the production of European sea bass, was also investigated by applying unsupervised PCA and supervised OPLS-DA to 1100–2500 nm NIR spectra [67]. PCA built to SNV-SG-second derivative spectral data did not return a clear separation of groups, mainly as a consequence of the fact that the intraclass variability among samples was higher than the among-class variability between samples. A correct classification rate of 100% for both wild and farmed sea bass was instead achieved by OPLS-DA, and, in this case, authors found VIP indexes related to proteins exerting a greater contribution to the variance between the two types of fish. A deeper insight into the different farming systems of aquaculture samples, moreover, showed the ability of NIR and OPLS-DA to authenticate 67%, 80%, 100% of extensively, semi-intensively, and intensively-reared subjects, respectively, thanks above all to the spectral bands associated with protein absorption [67]. Concrete tank-cultured sea bass were also successfully discriminated from sea cage-cultured sea bass during storage, by means of Vis-NIR spectroscopy coupled with PLS-DA [68]. The best performances (87% of correct classification), were observed for spectral measurements performed at 48 h post mortem [68]. However, the greater contributions of the wavelengths to the PLS discrimination of samples analysed at 48 h post mortem were different from those of samples analysed at 96 h post, thus classification by farming system may have been affected also by other unrelated factors, such as the well-known compositional changes occurring during shelf life.

Authentication by NIR and SIMCA analysis of European sea bass raised in extensive ponds, semi-intensive ponds, intensive tanks, and intensive sea-cages, was also performed both on fresh fillets and freeze-dried fillets [69]. Authors found that freeze-drying the samples gave the best classification outcomes. The same results were obtained when classifying fresh minced fillets and freeze-dried fillets of farmed European sea bass according to the semi-intensive conventional or the organic production system [70]. SIMCA classification based on second-derivative spectra (1100–2500 nm) of samples, in fact, generated good results when fitted on the freeze-dried fillets (65–75% of correct classification), and worse results when performed on fresh fillets (20–25% of correct classification) [70]. All these results are particularly informative about problems posed by water when analysing high-moisture foods like fish. One of the main drawbacks of NIR spectroscopy is, in fact, the difficulty in separating relevant from useless information from spectra, in which peaks of water are predominant. These peaks, when included in chemometric calculations may hinder reliable features related to functional groups of molecules of interest and, thus, produce misleading results, especially when samples only slightly differ, such as in the case of fish reared under different conditions.

Following these principles, NIR spectroscopy was also used to directly authenticate freeze-dried rainbow trout fillets by rearing farm and, at the same time, to check whether NIR discriminating capability changed between raw and cooked freeze-dried fillets [71]. Rainbow trout samples came from three different aquaculture systems, varying in average well water temperatures, of which one consisted in indoor rearing at 11–14 °C, one in outdoor rearing at 9–11 °C, and one in outdoor rearing at 3–14 °C. Results for classification by farm (using SNV and second derivative 1100–2500 nm spectra of raw samples) showed approximately 97–100% of accuracy, with k-NN analysis giving the best overall statistical performances and PLS-DA the worst ones. As for cooked freeze-dried samples discrimination, the accuracy was approximately the same as those obtained for raw samples (90–100% for LDA, QDA, k-NN and 80% for PLS-DA), highlighting that the cooking process did not alter the capabilities of the technique to discriminate the sample by rearing farm [71].

#### 3.2.2. Application of NMR Spectroscopy

Several applications of NMR spectroscopy aimed at authenticating the production process or the farming system were found in literature. In particular, proton (^1^H) NMR spectroscopy can be used to analyse lipid mixtures such as fish oil, requiring simple preparation of samples and short time of spectra acquisition and providing a great deal of useful information [72]. Thus, considering that fish flesh lipids are the main compounds changing on the basis of the feeding regime, many attempts to use ^1^H-NMR to identify the production process or the farming system were made. One of the earliest studies used SVM to elaborate ^1^H-NMR spectra, and it was highly effective in predicting the wild or the farmed origin of salmon from different European countries [72]. Similarly, encouraging results were achieved through the combination of ^1^H-NMR fingerprinting of lipids from gilthead sea bream with more complex chemometric data analyses [73]. The only unsupervised PCA applied on raw or processed ^1^H-NMR spectral profiles returned, in fact, a clear separation between wild and farmed samples, which was found to be linked to methyl and methylene protons, together with methylene and methyne protons in unsaturated fatty acids [73]. Moreover, LDA variables selection allowed classification of 100% of the tested wild and farmed samples, and results from probabilistic neural network (PNN) analyses further reinforced the findings that such class discriminations were readily feasible.

If the previous studies were performed on fresh raw fish, other studies were intended to evaluate any differences in classification outcomes deriving from various degrees of fish processing. Lipids extracted from different types of processed Atlantic salmon products (frozen, smoked, and canned) were subjected to ^1^H-NMR fingerprinting to develop models for determining labelling authenticity (wild/farmed) of these products [74]. SIMCA analysis applied to 138 pre-selected spectral peaks of NMR data, correctly classified as 100% of wild and 100% farmed samples, thanks mostly to the influence of a higher content of linoleic and oleic acid in farmed salmon compared to wild salmon [74]. A higher content of unsaturated fatty acids (and especially *n*−3 polyunsaturated fatty acids) was also found to play a special role in the discrimination between wild and farmed specimens of gilthead sea breams [75]. The influence exercised by these compounds was studied though the application of a supervised OPLS-DA to the whole lipid fingerprinting data obtained by ^1^H-NMR spectroscopy. Just like SIMCA classification did in the previous study, OPLS-DA also led to a perfect separation of samples, but with the great advantage of being able to highlight the most effective variables in discrimination in the simplest of ways.

The ^1^H-NMR molecular profiles of gilthead sea bream fish specimens produced according to different farming systems, have also been investigated, to seek out differences among three different kinds of aquaculture practices (cage, tank, and lagoon), but also any variations in the molecular patterns after a 16-day storage time under ice [76]. PCA-score plot of the pre-treated spectra showed a clear separation of fresh samples from ice-stored samples. At the same time, three distinct sub-clusters for each of the storage times, corresponding to the three farming systems investigated, highlighted the ability of the proposed methods to detect those molecular changes taking place during fish storage and exploited them for authentication purposes.

Another different NMR approach retrieved from the published literature concerned the use of carbon-13 (^13^C) NMR instead of ^1^H-NMR. Authors combined ^13^C-NMR spectra of muscle lipids of Atlantic salmon with PNN and SVM chemometric elaborations, to discriminate between farmed and wild samples and obtained excellent discrimination performances (98.5% and 100.0% of correctly classified samples, respectively) [77]. Despite ^13^C-NMR signals being generally much weaker than those provided by ^1^H-NMR (as well as time of analysis is often longer), useful and complementary information can be obtained by this technique.

### 3.3. Geographical Origin Falsification

Proving the geographical origin authenticity of fish and seafood often involves the use of multi-disciplinary and cross-disciplinary approaches which take account of the environmental and genetic backgrounds affecting fish final characteristics [78]. Several published scientific researches concerning the use of spectroscopic methods pointed out the usefulness in classification of fish and seafood according to country or FAO area of origin.

#### 3.3.1. Application of Vibrational Spectroscopy

Unlike the other authentication issues discussed above, NIR spectroscopy has been less explored for fish geographical origin identification. The reason, probably, is the great difficulty experienced in modelling total variability of NIR spectra and uniquely steering it to provenance, since provenance is the sum of a huge amount of different intrinsic or extrinsic factors (genetic, growth pattern, feeding regime, muscular activity, water temperature and salinity, etc.).

A traceability model able to predict the geographical origin of Chinese tilapia fillets coming from four different Chinese provinces, was developed by NIR spectroscopy [79]. SIMCA analysis, performed on 1000–2500 nm spectra of the minced samples, allowed more than 80% of fillets from Guangdong, Hainan, and Fujian provinces and 75% of fillets from the Fujian province to be correctly and exclusively assigned to the corresponding area of origin. Several locations in the Northern China Sea and East China Sea, from which sea cucumber (*Apostichopus japonicus*) come from, were also identified by using NIR spectroscopy [80]. In this case, authors found pre-treated (SNV or MSC, and second derivative) 1000–1800 nm spectra to give the best performance in PCA, since 100% correct classification rate was obtained both in the internal calibration model and in the external validation model. Similarly, 100% of sea cucumber analysed by means of diffuse reflectance MIR spectroscopy (fingerprint 5800–16,600 nm region) combined with SIMCA, were discriminated by the Chinese geographical region of provenance [81].

The last available application of NIR spectroscopy concerned the authentication of European sea bass according to Western, Central, or Eastern Mediterranean Sea provenances, by using OPLS-DA as a classification technique [67]. Results showed an overall discrimination performance of 89% according to these geographical origins, with 100% of Eastern, 88% of Central, and 85% of Western Mediterranean Sea samples being correctly classified. The VIP index analysis, moreover, identified lipid-associated bands as the most influential variables on the samples geographic discrimination.

#### 3.3.2. Application of NMR Spectroscopy

Masoum et al. (2007) proposed a method for the origin authentication of Atlantic salmon based on ^1^H-NMR and SVM of spectra extracted from samples coming from Canada, Alaska, Faroes, Ireland, Iceland, Norway, Scotland, and Tasmania. SVM returned a low degree of misclassification (4.6%) and, thus, an excellent correct classification rate for all the salmon samples [72]. Likewise, Aursand et al. (2009), used NMR combined with pattern recognition techniques to assess the geographical origin of Atlantic salmon and to verify the origin of market samples [77]. Here too, muscle lipids were extracted from tissues of fish coming from the same origins as those previously listed, but on the contrary, lipid composition was studied by ^13^C-NMR coupled with PNN or SVM. This time, although the PNN- and SVM-based approaches used returned different correct classification rates (93.8% and 99.3%, respectively), a comparable classification accuracy between the two methodologies approaches was observed [77]. The ^1^H-NMR lipid fingerprint, elaborated by LDA or PNN, allowed also to differentiate 76.2–100% of wild and farmed gilthead sea bream samples coming from Italy, Greece, Croatia, Turkey, and the Mediterranean Sea (for wild specimens), with better classification rates when PNN was applied [73]. Farmed gilthead sea bream specimens coming from five geographically distinct sites of Sardinia (Italy) and Greece were also discriminated by means of ^1^H-NMR lipid fingerprint [75]. In this case, the fraction of unwanted variability related to the different production system of samples (off-shore sea cages and lagoon) was successfully overlooked thanks to the application of the OPLS-DA and, although authors did not provide statistical outcomes from internal or external classification, the significance of the clusters observed in the score plot was confirmed by bootstrap statistical analysis. The highest bootstrap values (indicating a well-defined class separation) were obtained for discrimination between Greek and Sardinian fish (100%), while lower but meaningful bootstrap values were obtained for discrimination among samples coming from different Sardinian offshore sea cage farms (68–57%) [75].

One last interesting application of ^1^H-NMR dealt with the geographical authentication of bottarga, a fish-derived product consisting of salted and dried mullet (*Mugil Cephalus*) roe [82]. Low-molecular weight metabolites of aqueous extracts of samples, were analysed by PCA in order to identify clusters corresponding to one of the specific geographical provenances studied, namely FAO 37.1.3, FAO 34, FAO 41, FAO 31, and one unknown provenance. Results from PCA confirmed the possibility to characterise bottarga samples having different geographical origins, since samples with the same known geographical origin were closely clustered in the same region of the PCA scores plot, and those of different origin were far away from each other.

### 3.4. Discrimination between Fresh and Frozen/Thawed Fish and Seafood

Fish is commonly processed by freezing in order to be preserved from deterioration. Frozen fish, however, is usually characterised by much lower quality and commercial value compared to fresh fish. Therefore, fraudulent practices consisting in the substitution of fresh with frozen/thawed products are not uncommon events [83]. Considering that labelling of fish must state if the fish is fresh, frozen, or previously frozen (or refreshed), discriminating fresh from frozen/thawed products is one of the most important authenticity issues. The differentiation between fresh and frozen/thawed products is hampered by difficulties in detecting those tiny physical and chemical variations occurring during freeze storage, which, moreover, do not cause any perceptible organoleptic change [83,84]. Therefore, the rapid confirmation of fish freshness by spectroscopy has been widely studied during the last few years and several published researches are currently available.

#### 3.4.1. Application of Fluorescence and Vibrational Spectroscopy

Front-face fluorescence spectroscopy is one of the earliest spectroscopic techniques historically applied to differentiate fresh from frozen/thawed fish. It has been demonstrated that typical changes in fluorescence spectra of aromatic amino acids, nucleic acids, and nicotinamide adenine dinucleotide (NADH) occur during storage, as a consequence of several reactions involving free amino acids and carbonyl compounds of reducing sugars, formaldehyde (produced from trimethylamine oxide), and malondialdehyde (produced from oxidation of fish lipids during storage). Therefore, changes in fluorescence of fish samples may be considered as fingerprints for fresh and aged fish fillet identification [85]. The fluorescence emission spectra of tryptophan (305–400 nm) recorded directly on whiting fillets and elaborated by factorial discriminant analysis (FDA) led to correct classification rates of 62.5% and 70.8% in the calibration and validation set, respectively. NADH fluorescence spectra (360–570 nm), indeed, were found to have a higher potential to differentiate fresh from frozen/thawed products as they allowed to achieve 100% of correct discrimination for both calibration and validation set [85]. More recently, the same authors confirmed the success of a similar methodology in authenticating freshness of sea bass samples. Fluorescence emission spectra at 340 and 380 nm, elaborated by FDA, led to 94.87% of total correct classification rate [86]. Additionally, the elaboration of NADH fluorescence spectra by Fisher’s linear discriminant analysis, was stated as a reliable method to rapidly discriminate fresh and frozen/thawed large yellow croaker fillets, since 100% of total correct classification rate was achieved [87].

More applications of IR spectroscopy are reported in the published literature. Uddin and Okazaki (2004) used NIR reflectance spectroscopy on dry extract of horse mackerel specimens to evaluate freshness [88]. Both PCA (using 1100–2500 nm spectra) and SIMCA analysis (using only three selected wavelengths which were strongly related to protein content) successfully discriminated 100% of fresh and frozen/thawed samples. Thereafter, the same authors performed further investigations on fresh and frozen/thawed red sea bream by using Vis-NIR spectroscopy in the 400–1100 nm region [89]. In this case, raw spectra were used to build an LDA model, by which 100% classification accuracy in prediction was reached. PLS-DA of SG-smoothed spectra (670–1100 nm) of shrimps subjected to different treatments (including ice, water, and brine at various salt concentrations), also led to 100% of fresh and frozen/thawed samples to be authenticated [90].

Another study was directed to compare classification ability of Vis-NIR (380–1080 nm) and NIR (1100–2500) spectroscopy in authenticating fresh and frozen/thawed swordfish and, through the application of PLS-DA, it was found that in this case, Vis-NIR spectra gave better results in the external validation (≥96.7% of correctly classified samples) [91]. Although worse outcomes were obtained by only using the NIR region, the technique, combined with SVMs, also authenticated 93% of fresh and 83% of frozen/thawed sole (*Solea vulgaris*) samples [92]. Again, high accuracy (90%) and sensitivity (80%) in prediction were observed for the discrimination of fresh and frozen/thawed tuna sample by Vis-NIR spectral analysis (350–2500 nm) combined with PLS-DA [93], while better and more homogenous SIMCA prediction results were obtained when using MIR (2500–14,300 nm) instead of NIR (800–2500 nm) regions for the discrimination between fresh and previously frozen Atlantic mullet fillets [94].

Ottavian et al. (2013) proposed an interesting three-step approach based only on NIR spectra and latent variable modelling techniques to develop a species-independent classifier able to simultaneously discriminate between fresh and frozen/thawed fish and, remarkably, overall classification accuracy of the method ranged between 80% and 91%, based on the strategy adopted and the instrument used [94]. By contrast, the only MIR region was found to be useful for determining whether whiting fish fillets have been frozen/thawed: when FDA was applied to the 3300–3570 nm MIR subregion (usually related to fatty acids absorption), 87.5% of sample spectra in the validation set was correctly identified [95].

Finally, one single application of Raman spectroscopy to the authentication of fresh fish is now available [59]. Lipid fraction of fish from several species (horse mackerel, European anchovy, bluefish, Atlantic salmon, red mullet, and flying gurnard) was extracted from three samples batches (fresh samples, once frozen/towed samples, and twice frozen/thawed samples), and then collected by a Raman spectrometer along the 5000–50,000 nm spectral range and using a 785 nm laser exciting source. Chemometric analysis, performed by PCA, identified three different clusters in the score plot, each corresponding to one of the three batches of fish investigated [59].

#### 3.4.2. Application of Hyperspectral Imaging Spectroscopy

Discrimination between fresh and frozen/thawed cod fillet was studied by Vis-NIR/HSI, using both a handheld interactance probe and an imaging spectrometer (for automatic online analysis at typical industrial speeds) [96]. Spectra resulting from the two instruments were pre-treated (SNV and second derivative) and statistically analysed by applying the Rosenblatt’s perceptron linear classifier to the first and third principal component of the imaging data. Results showed that fresh cod fillets can be completely separated from fresh/thawed cod fillets using only a few wavelengths in the Vis region, mainly related to the oxidation of haemoglobin and myoglobin which occur during freezing/thawing [97]. Similarly, hyperspectral data from Vis-NIR/HSI (380–1030 nm) combined with least square-SVMs, returned an average correct classification rate of 91.67% for fresh and frozen/thawed halibut fillets [97].

#### 3.4.3. Application of NMR Spectroscopy

NMR spectroscopy is considered to be a useful and suitable tool for the discrimination of fresh from frozen/thawed fish, since NMR signals are sensitive enough to changes in water mobility and its interaction with other molecules [98]. NMR spectroscopy has been already widely exploited to identify the various modifications in fish tissues occurring during freezing and thawing of fish [99,100,101,102]; however, as far as we know, no application of this technique for fish freshness authentication is currently available.

## 4. Critical Aspects and Limitations to Overcome

The food scientists’ interest towards the development of reliable methods for the resolution of several food authenticity issues is well documented by the increasing number of scientific works which, albeit through different methodologies, have attempted to address the same problems. It is clear from the analysis of the latest literature that spectroscopy combined with chemometrics is just one of the many untargeted strategies adopted: chromatographic, MS-based, as well as bio-molecular and sensory techniques have been already widely exploited and have demonstrated their exceptional multipurpose qualities for fish authenticity testing [78,103,104,105,106,107,108].

These techniques are known to share certain common disadvantages, such as the long time needed for analysis, high costs of the equipment, the need of sample preparation prior to analysis, destructiveness, and the demand for qualified personnel. On the other hand, as they become more consolidated within the research community, these techniques excel by their higher accuracy, specificity, and sensitivity compared to spectroscopic ones, to the point that many of them are used in food official controls. Despite this, the attractiveness of spectroscopy and chemometrics is evidenced by not only by the large literature provided in the present review, but also by several other applications covering a wide range of food and foodstuffs: fruits and vegetables, honey, wine, edible oils and fats, cereal and cereal-based products, milk, and dairy products [109,110,111,112,113,114] have been successfully investigated and authenticated by means of spectroscopy.

Having said that, some critical reflections should be made about the problems related to the use of spectroscopy and chemometrics, which still have not been overcome. In accordance to what has been already reported and to our opinion, the research papers analysed were found to be highly variable to each other in terms of analytical set-up (e.g., sample pre-processing, spectral ranges, spectra pre-treatments, resolutions, number of samples tested, and statistical elaboration). This variability, as easily understood from Section 3, is further worsened by the fact that only a few of the works analysed reported in-depth statistical outputs and, where present, they were not comparable to each other.

A critical and objective evaluation of these works is also severely hampered by a lack, in certain cases, of comprehensive data with regards to the validation of the results. Alongside the internal cross-validation, the external validation of the qualitative chemometric model is, in our opinion, a crucial point in assessing the overall goodness of the classifiers and avoiding misleading interpretations. The last aspect which should be emphasised is that a detailed description of the characteristics of the sample dataset was not often reported and the lack of standardisation of external factors (e.g., storage times and conditions), may have interfered with spectral analysis, possibly affecting the robustness of the model. In this scenario, a recommendation for future works is to consider the intrinsically natural variability of the fish products (as well as those of all other foodstuffs), and to organise the sampling in such a way that as much of the expected variability of samples is collected during the calibration stage. That way, the robustness of the models can make their way to the spread of applications also in the industrial sector.

As a final remark, no technique should be universally regarded as the optimal solution. However, the possibility of using UV, IR, Raman, and NMR spectroscopies with no distinction for food authentication purposes is still an obstacle to overcome, and therefore, in accordance to our experience, untargeted NIR spectroscopy represents the most versatile option thanks to its high sensitivity to organic molecules of food, cost-effectiveness, and ease of use. Additionally, the use of NIR spectroscopy with supervised chemometric method, able to separate relevant from non-relevant spectral variation like OPLS-DA, should be encouraged since the interpretability of results is enhanced.

## 5. Conclusions and Prospects for the Future

Recent increases in the complexity and competitiveness of the fishery and seafood sectors, have resulted in the presence, on the international market, of a huge variety of fresh and processed products, but at the same time, have meant that the risk of fraud deriving from substitution among look-alike products is now exponentially higher than it was even a few years ago. Thus, ensuring the truthfulness of fish and seafood claims concerning their quality and origin, has become an exceptionally important topic, firstly with a view to enable consumers to make informed decisions.

The overview presented in this review clearly highlights the effective support provided by analytical approaches based on spectroscopy and multivariate data analysis for the evaluation and monitoring of fish and seafood products authenticity. Fluorescence, vibrational, NMR, and HSI spectroscopic applications have been discussed, with an accent on the trends toward their use for several authentication purposes. In this connection, IR spectroscopy has been the most exploited technique, especially in studies concerning species and fresh for frozen/thawed products substitutions. NMR, instead, has shown many applications in the field on the production method, farming system, and geographical origin identification. By contrast, Raman and HIS have provided very encouraging results in some fish authentication fields, but their overall potential has so far been largely ignored.

Rapidity, non-destructive nature, ease of use, and high-throughput measurements make the spectroscopic non-targeted approach an ideal tool for quality control operations, especially in the context of daily routine and screening analysis in the food industry, and as a possible substitute of traditional analytical techniques. Thanks to the technological development of the spectroscopic instrumentation, the availability of miniaturized and portable devices on the market is rapidly growing, and this will contribute to an additional growth of applications in the food sector. On the other hand, these analytical strategies in the official control of foodstuffs are still far from being effectively applied, largely due to the need of a strict validation to assure further reliability and robustness of results before implementation as standalone tools. For these reasons, standardisation of the working conditions, optimisation of the chemometric software, and creation of large databases for data-sharing and for encouraging greater cooperation between food scientists, represent important current research fields and future challenges to be faced.

## Figures and Tables

**Table 1:** Comparison of different spectroscopic techniques used for food authentication purposes: summary of the main characteristics.

Spectroscopic Technique		Wavelength Range (nm)	Interaction Light-matter	Basic Principle	Sensitive Compounds	Information Obtained	Applications	Possible Limitations
UV–Vis	UV	2 × 10^2^–4 × 10^2^	Absorption/emission	Electronic transitions	Double-conjugated bonds; isolated double, triple, peptide bonds; aromatic and carbonyl groups	Molecular structure	Qualitative/quantitative	Need of sample preparation pH and temperature interferences
	Vis	4 × 10^2^–7.5 × 10^2^						
IR^1^:	NIR	7.5 × 10^2^–2.5 × 10^3^	Absorption	Vibrations/rotations of molecular bonds (changes in dipole moments)	Polar bonds (N–H, C–H, O–H, S–H, C–O)	Chemical bonds and physical structure	Qualitative/quantitative	Water interferences Overlapping of spectral peaks
	MIR	2.5 × 10^3^–2.5 × 10^4^						
Raman		2.5 × 10^3^–1.0 × 10^6^	Scattering	Vibrations of molecular bonds (changes in polarizability)	Non-polar double or triple bonds (C = C, C ≡ C)	Chemical bonds and physical structure	Qualitative/quantitative	Fluorescence and photodecomposition interferences Low-intensity Peaks
HSI		Varying by spectroscopic modules	Absorption/emission/scattering	Varying by vibrational spectroscopic modules	Varying by vibrational spectroscopic modules	Varying by vibrational spectroscopic modules	Qualitative/quantitative/spatial	Varying by vibrational spectroscopic modules
NMR		5.0 × 10^8^–7.5 × 10^9^	Absorption	Nuclear spin changes	Nuclei having a proper magnetic field (spin quantum number ≠ 0 ^2^	Regio/stereo chemistry of molecules	Qualitative/quantitative/structural	Cost of the equipment

^1^ Infrared (IR) electromagnetic regions taken into consideration do not include far-infrared (FIR) range (2.5 × 10^4^–1.0 × 10^5^ nm) since it is not commonly used in food authentication studies. ^2^ H-1, C-13, and P-31 are the most frequently investigated nuclei in food science-related nuclear magnetic resonance (NMR) applications.

## Abbreviations

ANN	artificial neural networks;
BBN	Bayesian belief network;
FIR	far-infrared;
FDA	factorial discriminant analysis;
FFT	fast Fourier transform;
FT	Fourier transform;
HCA	hierarchical cluster analysis;
HSI	hyperspectral imaging;
IR	infrared;
k-NN	k-nearest neighbors;
LDA	linear discriminant analysis;
LW-NIR	long-wave near infrared;
MIR	mid-infrared;
NMR	nuclear magnetic resonance;
MSC	multiplicative scatter correction;
NIR	near-infrared;
OPLS-DA	orthogonal partial least square-discriminant analysis;
PCA	principal component analysis;
PLS-DA	partial least square-discriminant analysis;
PNN	probabilistic neural network;
QDA	quadratic factorial analysis;
SERS	surface-enhanced Raman spectroscopy;
SG	Savitzky–Golay smoothing;
SIMCA	soft independent modelling of class analogy;
SNV	standard normal variate;
SVM	support vector machine;
SW-NIR	short-wave near infrared;
UV	ultraviolet;
Vis	visible.
